# Understanding heterogeneity of responses to, and optimizing clinical efficacy of, exercise training in older adults: NIH NIA Workshop summary

**DOI:** 10.1007/s11357-022-00668-3

**Published:** 2022-10-15

**Authors:** Melissa L. Erickson, Jacob M. Allen, Daniel P. Beavers, Linda M. Collins, Karina W. Davidson, Kirk I. Erickson, Karyn A. Esser, Matthijs K. C. Hesselink, Kerrie L. Moreau, Eric B. Laber, Charlotte A. Peterson, Courtney M. Peterson, Jane E. Reusch, John P. Thyfault, Shawn D. Youngstedt, Juleen R. Zierath, Bret H. Goodpaster, Nathan K. LeBrasseur, Thomas W. Buford, Lauren M. Sparks

**Affiliations:** 1 Translational Research Institute, AdventHealth, 301 E Princeton St, Orlando, FL 32804 USA; 2 Department of Kinesiology and Community Health, University of Illinois at Urbana-Champaign, Champaign, IL USA; 3 Department of Statistical Sciences, Wake Forest University, Winston-Salem, NC USA; 4 Department of Social and Behavioral Sciences, New York University, New York, NY USA; 5 Institute of Health System Science, Feinstein Institutes for Medical Research, Northwell Health, New York, NY USA; 6 Department of Physiology and Functional Genomics, University of Florida, Gainesville, FL USA; 7 Department of Nutrition and Movement Sciences, NUTRIM School of Nutrition and Translational Research in Metabolism, Maastricht University, Maastricht, Netherlands; 8 Department of Medicine, Division of Geriatric Medicine, University of Colorado Anschutz Medical Campus, Aurora, CO USA; 9 Department of Statistical Sciences, Duke University, Durham, NC USA; 10 Center for Muscle Biology, College of Health Sciences, University of Kentucky, Lexington, KY USA; 11 Department of Nutritional Sciences, University of Alabama at Birmingham, Birmingham, AL USA; 12 Department of Molecular and Integrative Physiology, University of Kansas Medical Center, Kansas City, KN USA; 13 Edson College of Nursing and Health Innovation, Arizona State University, Phoenix, AZ USA; 14 Department of Physiology and Pharmacology, Karolinska Institutet, Stockholm, Sweden; 15 Department of Physical Medicine and Rehabilitation, Mayo Clinic, Rochester, MN USA; 16 Department of Medicine, University of Alabama at Birmingham, 1313 13th St. S., Birmingham, AL 35244 USA; 17 Birmingham/Atlanta VA GRECC, Birmingham VA Medical Center, Birmingham, AL USA

**Keywords:** Aging, Exercise, Response variation, Clinical efficacy

## Abstract

Exercise is a cornerstone of preventive medicine and a promising strategy to intervene on the biology of aging. Variation in the response to exercise is a widely accepted concept that dates back to the 1980s with classic genetic studies identifying sequence variations as modifiers of the VO_2_max response to training. Since that time, the literature of exercise response variance has been populated with retrospective analyses of existing datasets that are limited by a lack of statistical power from technical error of the measurements and small sample sizes, as well as diffuse outcomes, very few of which have included older adults. Prospective studies that are appropriately designed to interrogate exercise response variation in key outcomes identified a priori and inclusive of individuals over the age of 70 are long overdue. Understanding the underlying intrinsic (e.g., genetics and epigenetics) and extrinsic (e.g., medication use, diet, chronic disease) factors that determine robust versus poor responses to various exercise factors will be used to improve exercise prescription to target the pillars of aging and optimize the clinical efficacy of exercise training in older adults. This review summarizes the proceedings of the NIA-sponsored workshop entitled, “Understanding Heterogeneity of Responses to, and Optimizing Clinical Efficacy of, Exercise Training in Older Adults” and highlights the importance and current state of exercise response variation research, particularly in older adults, prevailing challenges, and future directions.

## Introduction

Aging is the greatest risk factor for an overwhelming majority of chronic diseases and geriatric syndromes. The conventional view that the aging process is not modifiable is being replaced by the Geroscience Hypothesis, which posits that biological aging per se is receptive to intervention [1]. A current and unmet need in the field is defining therapeutic strategies that effectively intervene on the negative consequences of aging. Exercise is one of the most promising interventions to directly counter the biology of aging, delay physiological decline, and increase health span. A key opportunity for understanding how to best use exercise to improve health in older adults is to identify the underlying factors that contribute to variation in responsiveness to exercise across a variety of clinically relevant endpoints. This information will enable more precise and efficacious exercise prescriptions to optimize the clinical efficacy of exercise training in older adults.

**Fig. 1:**
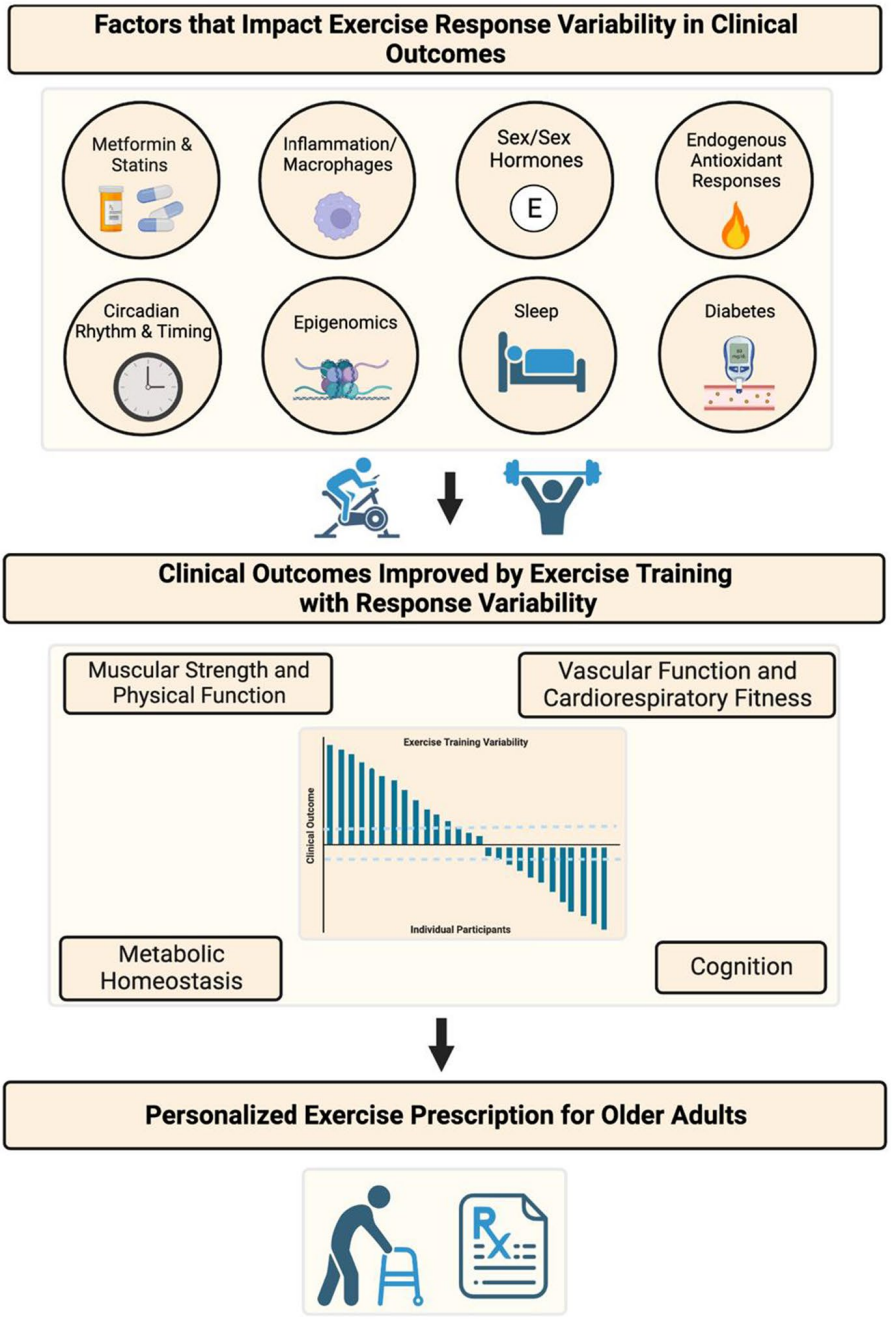
Schematic summarizing factors identified by the NIH NIA Workshop that contribute to exercise response variation in outcomes clinically relevant for older adults. Waterfall plot is a theoretical depiction of summary data from a controlled exercise training study. Each bar represents the delta (pre- to post-exercise training) from individual participants for a clinically relevant outcome modified by exercise training, and dashed lines represent technical error

**Fig. 2:**
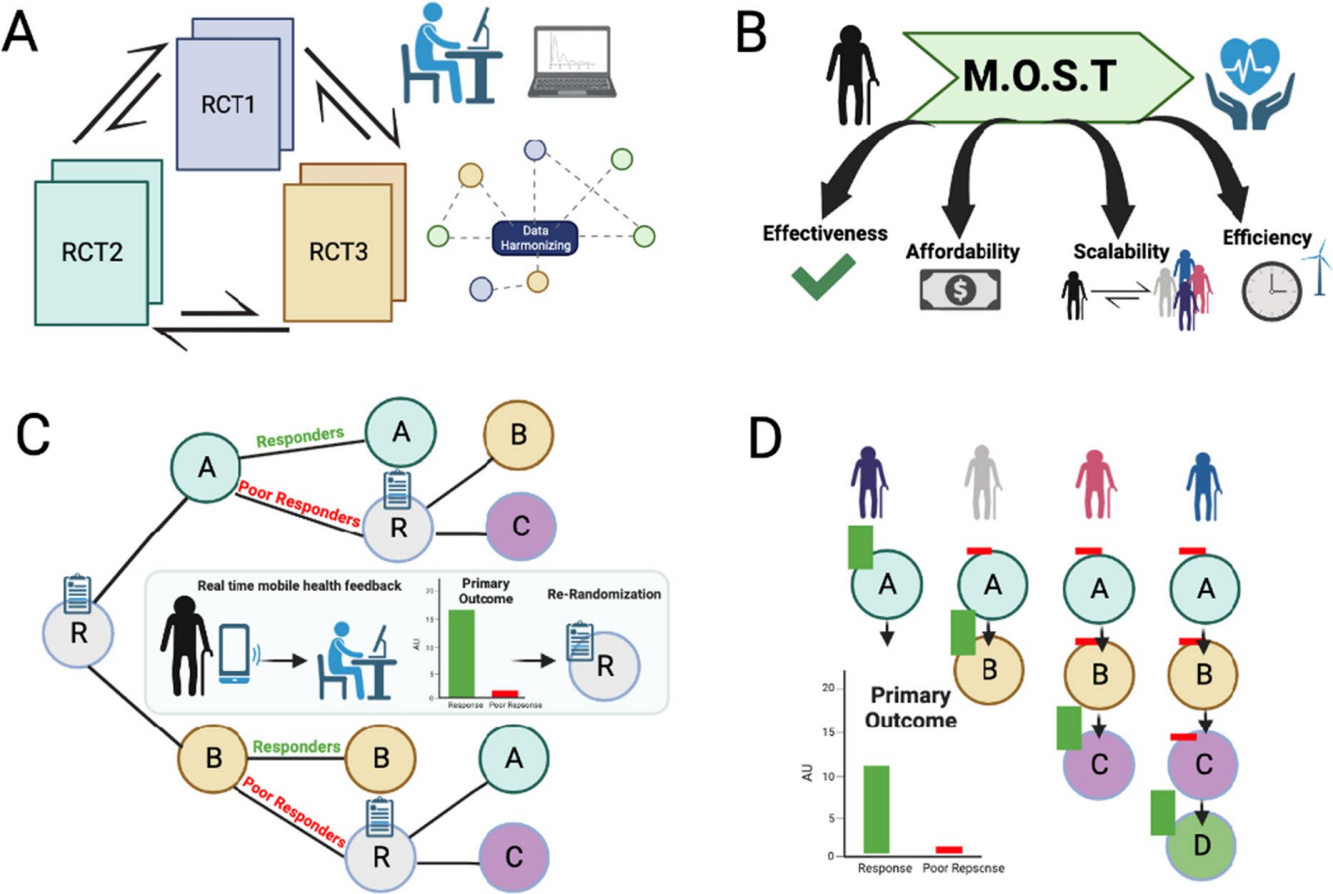
Summary of four (4) alternative study design strategies identified by the NIH NIA Workshop. **A** Pooled randomized controlled trials. **B** Multiphase optimization strategy (MOST). **C** Micro-randomized and SMART trials. **D** Personalized N-of-1 trials. These study designs may be adapted by researchers to better understand exercise response variation in aging populations

This review summarizes discussions from the workshop entitled, “Understanding Heterogeneity of Responses to, and Optimizing Clinical Efficacy of, Exercise Training in Older Adults: NIH NIA Workshop Summary,” which was held virtually on April 7–8, 2022. The primary goal of this workshop was to identify significant modulators, mechanisms, and biomarkers that explain exercise response variation in endpoints that are clinically relevant for older adults. Herein, we summarize evidence for factors that impact exercise response variation on clinical outcomes modifiable by exercise training including (A) muscular strength, hypertrophy, and physical function; (B) vascular function and cardiorespiratory fitness; (C) metabolic health, such as glycemic control, body weight, and gut microbiome; and (D) brain, sleep, and cognitive outcomes (Fig. 1). We identify key research gaps and transdisciplinary approaches for understanding intrinsic and extrinsic factors that underlie variations in exercise response, particularly in older adults. For example, it may be possible to identify those who are more likely to have poor physiologic responses to an exercise intervention. We may also learn what factors underlie better responses to exercise; and the extent to which these factors are modifiable may improve clinical efficacy of the exercise. Another important aspect for consideration in research on exercise response variation is clinical trial design. For example, the field of personalized medicine uses study designs other than randomized controlled trials to address individual response variation, and these approaches may be relevant for optimizing clinical efficacy of exercise training interventions in a heterogenous population, such as older adults (Fig. 2). Ultimately, gaining this knowledge will support our goal of improving the efficacy of exercise prescription for all older adults by countering biological aging and increasing health span.

## Heterogeneity in biological aging

Numerous conditions and diseases are associated with the biology of aging, inclusive of but not limited to musculoskeletal conditions and cardio-metabolic dysfunction. As the obesity epidemic coincides with aging, increased vulnerability to cancers, neurodegeneration, and cardiovascular dysfunction, as well as frailty and disability, is also on the rise. The underlying molecular changes that occur during aging are commonly summarized into the following seven pillars: (1) changes in adaptation to stress, (2) inflammation, (3) macromolecular damage, (4) metabolic dysregulation, (5) proteostasis, (6) stem cell regeneration, and (7) epigenetic drift [2]. A current unmet public health need is identifying interventional strategies that delay the onset of these conditions by directly targeting the pillars of aging. A challenging factor, however, is that the biology of aging itself is heterogeneous. Thus, the clinical efficacy of exercise training in older adults must be interpreted within the context of biological aging heterogeneity.

Physiological adaptation to chronic exercise improves several age-related health outcomes, highlighting the probability that exercise itself positivity impacts the fundamental biology of aging. For example, exercise profoundly impacts the musculoskeletal, cardiovascular, metabolic, cognitive, immune, kidney, and pulmonary systems. Exercise also beneficially impacts age-related DNA damage, mitochondrial dysfunction, proteotoxic stress, and inflammation by preventing senescence, facilitating cell clearance, reducing DNA damage, and improving mitochondrial function [3, 4]. Exercise and physical activity improve endurance, strength, balance, and flexibility, and are therefore well-accepted strategies to promote healthy aging. The crux of this issue, and the impetus for convening this NIA Workshop, is that while exercise has been consistently shown to impart the aforementioned improvements in numerous aspects of health—not all individuals reap benefits from exercise in an equivalent manner. Moreover, how the confluence of various clinical and biologic inter-personal factors converges to influence these outcomes is, at present, poorly understood. Thus, an improved understanding of the causes of, and potential therapeutic strategies to overcome, exercise response heterogeneity is an urgent research need with important implications for impacting public health.

## Exercise response variation

Individual response variation to a given dose of exercise within studies was reported in the scientific literature nearly 40 years ago [5–10]. As a result, the Health, Risk Factors, Exercise Training, and Genetics (HERITAGE) Family Study sought to address the genetic contributions of changes in cardiorespiratory fitness (VO_2_max) in response to a standardized exercise training program. The most recent estimates suggest that interindividual variation in VO_2_max ranges from − 4.7 to 47.8%. Moreover, the largest contributing factor to this variation was genetic heritability, explaining 47% [11]. In addition to heritability, other factors influencing the change in VO_2_max following exercise training response included baseline VO_2_max, age, sex, weight, and ethnicity [11]. In older adults, exercise positively impacts numerous clinically relevant outcomes in older adults by reducing fall risk, cardiovascular disease, and death. Since HERITAGE, several additional intrinsic (non-modifiable) and extrinsic (modifiable) factors that contribute to exercise response variation have been identified [12]. Understanding how these factors interact with the biology of aging will enable the optimization of clinical exercise prescriptions for older adults in the future.

Differences in the exercise stimulus also contribute to exercise response variation. Factors such as exercise mode, type, intensity, duration, time of day, and length of intervention influence expected adaptation responses. This point is further highlighted by a previous review summarizing research strategies to increase individualization of exercise prescription [13]. Another factor that impacts the magnitude of expected exercise adaptations is the central goal of a research study and recognizing that an improvement (or lack thereof) in the intended outcome may not equal a response in another outcome [12, 14]. For example, efficacy trials seek to determine whether an intervention produces the expected result in ideal circumstances. These trials use tightly controlled interventions, strict compliance criteria, and narrow inclusion/exclusion criteria. In contrast, effectiveness trials are more pragmatic, and commonly accept trade-offs between strict experimental control for real-world applicability and generalizability. Another aspect that is crucial to setting expectations for the magnitude of change induced by exercise is behavioral factors outside of a structured intervention. This may include, but is not limited to, how structured exercise impacts physical activity and sedentary behavior outside of interventions, the timing of meals and exercise, as well as duration and quality of sleep [15]. Furthermore, the selected research outcome of interest also contributes to response variation both within [11] and between studies.

Experimentation itself contributes to variation in measured outcomes. Measurement error is the variation of the measurement or methodology itself; thus, a meaningful biological change must exceed measurement error to be detected. Technical error adds another layer of interpretive complication, combining both measurement error and day-to-day variation [16]. Measurement and technical variation occur at random and, realistically, are difficult to avoid in real-world experimentation [17]. In the past, researchers have used different approaches for interpreting meaningful biological signals against experimental error, and this has led to inconsistences in the interpretation of biological trainability [16, 18, 19]. At present, there is no consensus on the “ideal” analytical approach to studying exercise response variation which is a critically unmet need that must be addressed.

## Knowledge gaps in aging and exercise response variation

The Molecular Transducers of Physical Activity Consortium (MoTrPAC) was designed to interrogate exercise response variation at the molecular level in healthy adults and children and is currently underway. While these data will shed novel insight into the exercise response variation through an -omics lens, MoTrPAC will not comprehensively assess clinical phenotyping data beyond muscular strength, VO_2_max, and body composition [20], leaving several knowledge gaps that need to be addressed in future work—particularly understanding how exercise may be used to intervene on the pillars of aging. Importantly, the degree to which exercise training adaptations are affected by the type and extent of age-related damage is unknown and, thus, several questions arise. How do different forms of age-related damage contribute to exercise response variation? What is the critical type of damage that is altering the health and function of these different tissues and the responsiveness to exercise? Is it the type, extent, or magnitude of age-related damage that is contributing to variation? What are the critical features that impact exercise responsiveness? How does the biology of aging influence the ability of cells to mediate exercise adaptations? Do differences in daily physical activity and sedentary behavior habits prior to the exercise intervention impact exercise adaptations? In addition, defining aging cut-offs and aligning these cut-offs with when the biology of aging is detectable remains challenging. Furthermore, fundamental questions such as the role of ethnicity, sex, and hormonal status are poorly understood. In addition to aging biology itself, there is evidence for several factors that contribute to exercise response variation. The following sections summarize the evidence provided by NIH NIA Workshop presenters on factors that contribute to exercise response variation in outcomes clinically relevant for older adults, followed by key knowledge gaps.

### Muscular strength, hypertrophy, and physical function

Aging is associated with declines in muscle mass, strength, and physical function, as well as increases in major mobility disability (MMD). Exercise training benefits older adults by directly targeting these age-associated changes. For example, progressive resistance training (PRT) increases muscle mass, fiber size, and strength [21, 22]. Similarly, structured moderate intensity, multi-modal physical activity reduces MMD in older adults at risk for disability [23]. Previous investigations of PRT [24] and multi-modal physical activity [25] have shown individual response variation, and the use of concurrent pharmacotherapy may be a contributing factor. Pharmacotherapy is often part of the established prevention or treatment strategy for several age-related diseases. For example, prescription trends for metformin, statins, and antihypertensives are on the order of millions, each reaching the top 10 most common prescriptions in 2019 [26]. Routine pharmacotherapy alters the cellular environment, and this may either amplify or blunt the beneficial adaptations to exercise training.

#### Metformin and statins alter progressive resistance exercise training adaptations in older adults

Exercise response variation to resistance training has been observed, including some poor responders, and this may be related to age-associated increases in muscle-specific inflammation [27]. Metformin is the first-line therapy for type 2 diabetes (T2D) prevention and is one of the most commonly prescribed drugs worldwide [28]. Metformin alters the cellular environment by promoting the anti-inflammatory actions of macrophages [29, 30], as well as inhibits the activity of parts of the electron transport system [31–33]. The MASTERS trial was designed to evaluate if metformin augments the response to PRT by reducing age-associated muscle inflammation in a cohort of adults aged 65 years and older [34]. The primary finding of the MASTERS trial was that metformin negatively impacted the exercise-induced increases in body mass and leg muscle mass, compared to placebo. Deeper molecular analyses revealed that, although metformin induced the expected changes in the macrophage environment, strength adaptions were blunted by metformin. Further investigation into combination pharmacotherapy points towards additional interactions with statins: participants on either metformin or statins alone tended to gain less muscle size, whereas those on the combination of metformin and statins gained nearly as much muscle mass as the placebo group. These muscular gains were associated with increased M2 macrophage abundance, suggesting inflammatory status contributes to physiological adaptations to PRT [35]. Overall, the results from the MASTERS trial contribute to our understanding the exercise response variation occurs in outcomes clinically relevant for older adults: muscular strength and hypertrophy.

#### Statins blunt exercise capacity

Statins are commonly prescribed to reduce cardiovascular disease (CVD) risk following a major event [36], and are gaining attention as a primary prevention tool. However, the use of statins in disease-free populations remains controversial due to several associated negative side effects, including pain, exhaustion, fatigability, muscle damage, myopathy and injuries, mitochondrial abnormalities, insulin resistance, and increased T2D risk [37, 38]. A controlled exercise study found that statins blocked exercise adaptation in previously sedentary individuals, assessed as changes in absolute and relative VO_2_ max, compared to an exercise only group [39]. Deeper analysis of skeletal muscle biopsy tissue from these participants showed that statins blunted improvements in mitochondrial content (citrate synthase), compared to the exercise only group that increased by 13%. Findings from preclinical studies on the combined use of statins and exercise are consistent with this human trial. For example, in animal models, atorvastatin blunted exercise training adaptations (assessed as distance to exhaustion). Tissue analysis showed that statins blunted improvements in skeletal muscle mitochondrial respiration [40, 41]. These findings raise the question of compatibility between statins and physical fitness, and there is some evidence that they can co-exist. Cross-sectional studies in older adults examining the intersection of fitness levels and statin use found that those on statins with the highest fitness also had the greatest level of protection against early mortality, whereas those not on statins with the lowest fitness had the greatest level of risk [42].

#### Anti-hypertensive medications impact late life physical function

Physical exercise is commonly considered the standard intervention for improving physical function among older adults. However, the extent of functional benefits from exercise is variable, with many individuals obtaining sub-optimal benefits despite strong adherence to exercise [43]. Thus, exercise appears to be a necessary component of treatment regimens to prevent age-related loss of physical function, but further refinement and personalization in the prescription is necessary [44]. Extensive evidence suggests that antihypertensive medications—particularly those which mediate the renin-angiotensin system (RAS) may influence functional outcomes [45]. Thus, the choice of first-line antihypertensive medications for older adults with hypertension may have an important role on functional responses after exercise. Retrospective analyses from the LIFE Trial showed that participants on ACE inhibitors (ACEis) may have additional benefits: a sample of 424 adults aged 70–85 years on ACEis had greater exercise-induced benefits in physical function compared to those taking other classes of hypertensive medications, as well as non-users [46]. The ACES trial is an ongoing trial currently evaluating the potential interactions of first-line antihypertensives with aerobic exercise [47], but future studies will need to evaluate interactions with resistance exercise as there may be differences in the drug-exercise interaction between exercise modes [48]. Taken together, there is evidence for interactions between exercise training and anti-hypertensive pharmacotherapy on physical function outcomes, although a mix of medication classes complicates interpretation.

#### Knowledge gaps

Given the widespread use of metformin, statins, and anti-hypertensive medications in older adults, the depth of knowledge on how exercise and drugs interact to impact muscular and physical function outcomes, as well as CVD and all-cause mortality risk, is severely lacking. Demographics such as age, sex, BMI, existing co-morbidities, and other drugs will affect hepatic uptake and catabolism (clearance) of drugs that are cleared by the liver (statins) or affect kidney function for drugs cleared in the urine (metformin). Metabolism and clearance of these drugs impact exposure of other tissues like skeletal muscle that can compromise function. Thus, commonly used pharmacotherapies have a high likelihood of contributing to exercise response variation. Studies that address the fundamental features of pharmacokinetics, such as dose, duration, type, and time of initiation, are desperately needed, together with clinically relevant outcomes for older adults that impact their quality of daily life and risk for future disease and death. Outcomes such as muscle size (e.g., cross-sectional area from biopsy, thigh muscle area by computed tomography scan, fat free mass by dual X-ray absorptiometry), muscular strength, and physical function outcomes (6-min walk test and the short physical performance battery, and assessments of cardiorespiratory fitness) are critical for maintenance of healthy aging, independent living, and overall quality of life. In addition to clinical studies, the field needs deeper mechanistic and molecular work in preclinical models to better understand how exercise interacts with metformin, statins, and anti-hypertensive medications that can be translated into more refined prospective clinical trial in older adults.

### Vascular function and cardiorespiratory fitness

Advancing age is the greatest risk factor for CVD [49]. Age-associated changes in the vasculature, such as arterial stiffening and endothelial dysfunction, are likely physiological drivers of CVD [50]. Cardiorespiratory fitness is another relevant outcome, as it is related to mortality, risk for several chronic disease conditions, and is negatively impacted by age-associated declines in vascular function. Regular exercise training improves both vascular function [50] and cardiorespiratory fitness at any age, and is thus a key therapeutic strategy to reduce CVD risk. While vascular health and cardiorespiratory fitness are both improved by exercise, the magnitude of exercise-induced adaptations varies by sex, age, as well as co-existence of age-related diseases such as T2D.

#### Age-related changes in sex hormones impact exercise-induced improvements in vascular endothelial function

Sex- and age-related changes in sex hormones modulate vascular endothelial adaptations to exercise training. Studies in humans show that regular endurance exercise enhances endothelial function assessed via brachial artery flow-mediated dilation (FMD; macrovascular function) and forearm blood flow response to acetylcholine (microvascular function) in older men, however, similar training benefits are diminished or absent in postmenopausal women [50]. Because sex hormones modulate vascular aging, sex-related differences in endothelial adaptations to endurance exercise may be related to the marked, relatively abrupt reduction in circulating estrogens with menopause in women, whereas a parallel change is not observed in men. In previously sedentary postmenopausal women, endothelial function (measured by FMD) increased following moderate intensity exercise training in women treated with estrogen but not in women treated with placebo [50]. Thus, estrogen may “recouple” or reconnect the exercise signal to permit endothelial adaptations to exercise training.

Chronic endurance exercise training benefits endothelial function by enhancing antioxidant defense systems, effectively lowering reactive oxygen species (ROS) bioactivity, and increasing resistance to oxidative damage in the vasculature. The favorable effects of endurance exercise training on lowering ROS production and vascular oxidative stress appear to be sustained with aging, at least in men. Infusion of vitamin C—a potent antioxidant—improved FMD in untrained older men but had no effect in exercise-trained older men, suggesting that exercise training benefits endothelial function by mitigating the effects of oxidative stress by reducing excessive ROS production and its tonic suppression of endothelial function [51]. On the other hand, vitamin C infusion increases FMD in estrogen-deficient postmenopausal women regardless of training status. In contrast, there is no effect of vitamin C infusion on endothelial function following endurance exercise training in postmenopausal women treated with concurrent estrogen. These data suggest that in the absence of high circulating estrogen, endurance exercise training has no obvious effect on vascular oxidative stress and its tonic suppression of endothelial function in postmenopausal women, whereas in the presence of high circulating estrogen, oxidative-stress related suppression of endothelial function is ameliorated with endurance exercise training [52]. Taken together, these studies suggest that endurance exercise enhances endothelial function in older men through improvements in antioxidant defenses, reduced ROS, and increased resistance to oxidative stress, whereas in postmenopausal women declines in estrogen during menopause reduce vascular adaptations to endurance exercise training, possibly due to a failure of antioxidant defenses to adapt to endurance exercise and mitigate vascular oxidative stress. This is striking, as the benefits of exercise on the vascular system are critical to overall health benefits of exercise. These data suggest that our expectations of exercise adaptations, particularly as it relates to vascular outcomes in older adults, should consider changes in sex hormones and oxidative stress when interpreting exercise response variation.

#### Type 2 diabetes is associated with reduced cardiorespiratory fitness

The presence of T2D itself may blunt the beneficial adaptations of exercise training to improve cardiorespiratory fitness [53]. Cross-sectional studies show that both adults [54] and youth [55] with diagnosed T2D have a robust decrease in functional exercise capacity. While the underlying mechanisms are still unknown, nitric oxide (NO) and nitric oxide synthase (NOS) have been implicated to play a role given that these molecules dually stimulate both mitochondrial biogenesis and dynamics in skeletal muscle while also modulating endothelial function in the vascular system [56]. Studies in preclinical models suggest that modulating endothelial NOS restores exercise training adaptations. For example, saxagliptin, an inhibitor of DPP4, a circulating enzyme that catabolizes glucagon-like peptide 1 (GLP-1), improved exercise-induced adaptations in endurance capacity in insulin-resistant rats compared to a no treatment control group [57].

Translational work in humans suggest that reduced oxygenated perfusion in cardiac and skeletal muscle tissue [53] underlies exercise intolerance that is associated with T2D. For example, cross-sectional studies in women with T2D find that participants maintain cardiac output but exhibit increased vascular resistance putatively leading to reduced blood flow to working skeletal muscles during exercise [58]. Furthermore, studies using magnetic resonance spectroscopy show that participants with T2D have impaired in vivo mitochondrial capacity, assessed as slower phosphocreatine recovery and adenosine triphosphate phosphate (ATP) production [59–61]. Interestingly, *in vitro* assessments of skeletal muscle mitochondrial function were not impaired, and this implicates a role for reduced skeletal muscle tissue perfusion as a characteristic of T2D [61] that also contributes to exercise impairments. Sex may also be relevant for phenotypic differences, as women with T2D have lower insulin sensitivity than men even after correcting for body mass [62]. Furthermore, women with T2D, as well as obesity, have lower-end diastolic and systolic cardiac size and function [58].

#### Knowledge gaps

To date, there are limited data on the role of estrogens or other hormones, including testosterone, on cardiovascular responses to exercise training in both men and women, respectively. At present, estrogen treatment is not medically indicated for CVD prevention in postmenopausal women. Learning more about the influence of age-related changes in sex hormones may inform strategies that directly target the pillars of aging and enable more people to benefit from the positive effects of exercise. Specifically regarding T2D and impaired adaptations to exercise, there are several unanswered questions. It is unclear why women have worse insulin resistance relative to men, and how T2D status and duration may impact these sex differences. An emerging hypothesis is that ambient hyperglycemia blunts favorable exercise adaptations in cardiometabolic outcomes [63]. Further research efforts should focus on testing if exercise can overcome microvascular dysfunction in those with T2D or if intensive pharmacological treatment to lower hyperglycemia (insulin for example) prior to exercise interventions improves outcomes.

### Metabolic homeostasis

Metabolic dysfunction is one of the pillars of aging [2], and both regular exercise and regular inactivity directly impact this pillar. Exercise is a well-accepted strategy to improve glycemic control in people with T2D [64]. Furthermore, lifestyle modifications, including exercise, is the frontline treatment for T2D. There is evidence that epigenomics, circadian rhythm, exercise, and meal timing, as well as the gut microbiome, contribute to exercise response variation in outcomes that drive metabolic health. The following section summarizes evidence indicating that these factors contribute to exercise response variation outcomes, including HbA_1C_, free-living glycemic control, peripheral insulin sensitivity, and other indices of cardiometabolic health.

#### Epigenomics underpin exercise response variation in type 2 diabetes risk factors

Both early and late life stimuli are capable of creating a metabolic environment that leads to epigenomic programming or “memory” in tissues. One recent example of this is the observation that low birthweight infants have higher liver fat and earlier onset of prediabetes compared to normal birth weight counterparts [65]. These findings suggest that differences in metabolic health across various stages of life have residual effects. For example, exercise response variation in a myriad of clinically relevant metabolic outcomes in individuals with T2D has been linked to baseline differences in transcriptional profiles in skeletal muscle, whereby super responders had 70% more upregulation of mitochondrial-related gene expression compared to poor responders [66]. In this case, super responders vs. poor responders were distinguished by changes in HbA_1C_, percent body fat, BMI, and skeletal muscle mitochondrial DNA in response to a 9-month supervised exercise intervention. Myogenetic progenitor cells retain the metabolic phenotype of the donors enabling *in vitro* investigations of cell autonomous contributions to exercise response variation. In this model, baseline differences in epigenomic profiles are linked to exercise response variation in clinical risk factors in individuals with T2D [12]. Taken together, these studies highlight the importance of considering how the metabolic milieu across the lifespan contributes to epigenomic programming of skeletal muscle (and other tissues), which in turn impact the magnitude of adaptation to standardized exercise interventions.

#### Knowledge gaps

The role of epigenomics is not well understood. There is a critical need for larger cohorts, as well as more robust epigenomic profiling, such as chromatin accessibility and histone modifications. Another key knowledge gap is addressing the rate of response to an exercise intervention. In other words, the time course of adaptations to exercise training may differ between individuals, and this variation is missed by study designs that use single timepoint post-test assessments (e.g., only one post-test assessment after 12 weeks of controlled exercise training). Between and within-subject variation in VO_2_max rate of response to controlled exercise training has been demonstrated in healthy, middle aged-adults using a study design with longitudinal VO_2_max assessments, in 3-month increments, across a 1-year [67]. Rate of response variation is an established concept in the weight loss field. For example, fast and slow responders during weight loss interventions have been previously identified and may be caused by genetic differences [68, 69]. To further complicate matters, the variation of rate of response may differ by outcome. For example, exercise adaptation rates to VO_2_max may differ compared to a glycemic outcome, such as HbA_1C_. In reference to exercise training in older adults, this central question remains: does age-related damaged contribute to a slower exercise adaptation? Further studies should seek to address this, as well as to determine broader role of aging biology itself, as a contributor to exercise response variation in clinically relevant outcomes.

#### Time-of-day of exercise modifies the metabolic responses to exercise

Circadian clock mechanisms exist in virtually every cell, functioning as endogenous molecular timekeepers by regulating a daily program of gene expression. A consequence of the intrinsic clock mechanism is that the response to a physiological stimulus varies depending on the time of day. This has been shown with exercise in preclinical models, in which the transcriptional response to a single bout of running elicited diverse responses [70, 71]. Furthermore, acute exercise in rodent models at either the early active phase or late active phase differentially impacts the skeletal muscle metabolome, as well as systemic energy expenditure [71]. Exercise and nutrition interventions may also be synchronized to circadian timing for optimal free-living blood glucose control. A controlled exercise study in men found that the same regimen of high intensity interval training was more efficacious for controlling free-living postprandial glucose when performed in the afternoon, compared to the morning [72]. These findings complement a retrospective analysis that compared morning vs. afternoon exercise training for 12 weeks in men at risk for T2D. These comparisons showed that those who completed exercise training in the afternoon had 13% larger improvements in glucose infusion rates, compared to morning exercisers [73]. In addition, afternoon exercise training also led to greater declines in fat mass compared to morning exercise training. Taken together, these early studies point to circadian biology and exercise timing as factors that contribute to exercise response variability in metabolic health. The role of meal timing in relation to exercise is likely pertinent, and future studies should consider various exercise and meal timing schemes.

#### Knowledge gaps

Despite the pervasive nature of the circadian system, controlled studies in chronobiology and exercise outcomes are lacking. Fundamental questions such as the impact of age, sex, and ethnicity on circadian clocks, circadian phase response in muscle and other peripheral tissues, and clock output (downstream changes in gene expression) are currently unknown. To better understand the muscle transcriptome, more experiments using women, different ethnicities, ages, and stages of pathogenesis, are needed. There is limited information on circadian variation in proteomics, post-translational modifications, and metabolomics, highlighting a need for interrogative -omics approaches in exercise circadian biology. The majority of studies to date focus on transcriptomic analysis of muscle, yet several organs are involved in metabolic homeostasis. We do not yet understand how proteins change in a diurnal manner, or how proteins are modified throughout the day. Furthermore, the role of metabolic disease in altering metabolic rhythms is unknown. The internal communication between circadian clocks across peripheral tissues (e.g., circadian alignment between the liver and muscle clocks) may also be relevant in context of metabolic function. Taken together, the optimal exercise intervention for metabolic health is unknown. Acute vs. chronic exercise training, training in alignment with biological rhythms to maximize adaptations, as well as synchronizing exercise with nutritional status to amplify or blunt metabolic adaptations, are topics that have not been addressed.

#### Nutrient timing may modulate the cardiometabolic effects of exercise training

Nutrient timing and sensing may contribute to the variation in exercise response, particularly as it relates to outcomes relevant for cardiometabolic health. The field of human aging research has long recognized nutrition as an important modulator of the aging process. The Comprehensive Assessment of Long-Term Effects of Reducing Intake of Energy (CALERIE) Study showed that ~ 15% caloric restriction, without malnutrition, slows metabolic rate in non-obese adults and improves several aging-related outcomes [74]. Nutrient timing is now emerging as a novel dietary aspect of longevity. Preclinical models show that at least 40% of the life-extending benefits of calorie restriction are instead due to the extended fasting duration rather than energy restriction per se and eating out of alignment with circadian rhythms attenuates these effects [75, 76]. This concept of nutrient timing is complex and there are several aspects to be considered, such as the fasting duration (e.g., pre- versus postprandial state, number of hours of fasting), time-of-day of food intake, consistency of mealtimes, nutrient composition (i.e., macronutrients, glycemic index), and meal frequency. In addition, how these nutrition/energy intake factors relate to the timing of exercise is also critical. Preliminary data suggest that exercising in the pre-prandial state before breakfast increases fat oxidation, improves glucose tolerance, and may facilitate weight loss in adults without type 2 diabetes [77–79]. Further, the fasting duration impacts substrate metabolism; for example, fasting for 14–20 h increases whole body fatty acid oxidation and hepatic gluconeogenesis [80]. Since fasting duration alters substrate availability and myriad downstream pathways, the number of hours of fasting could affect exercise responsiveness. It is currently unknown whether individuals who are less metabolically flexible (such as those with obesity) receive the same benefits for the same fasting duration as metabolically flexible individuals. The time-of-day of food intake and individual circadian rhythms may also influence exercise response. Front-loading calories to earlier in the day has been shown to improve weight loss and indices of cardiometabolic health, including insulin sensitivity [81, 82]. This is likely explained by diurnal rhythms in metabolism, particularly in glycemic control and the thermic effect of food, which tend to peak in the mid to late morning in most individuals [83]. However, there is heterogeneity in the amplitude and phases of circadian rhythms and other diurnal rhythms, which could lead to heterogeneity in the optimal timing of both food intake and exercise. In particular, adults with obesity may have weaker circadian rhythms [84], which has been linked to slower weight loss [85]. Consistency in the timing of food intake (regular or irregular) may also contribute to intervention variation. A retrospective analysis of CALERIE found that consistent meal timing was associated with better weight loss [86]. Since exercise also entrains circadian rhythms, consistency in the timing of exercise may influence outcomes relevant to aging. Finally, meal frequency and the timing of macronutrient intake across the day (e.g., the time of day when one consumes carbohydrates or proteins) may affect health outcomes; however, less is known about these topics. There are parallel factors with exercise; more specifically, exercise frequency and the timing of types of different exercise modalities (i.e., resistance vs. aerobic training) may account for some of the heterogeneity in exercise response.

#### Knowledge gaps

It is unclear which aspects of nutrient and/or exercise timing have the biggest impact on cardiometabolic health and what are the underlying molecular sensing and physiological mechanisms. It is critically important to uncover which aspects of this biological heterogeneity are malleable and modifiable (e.g., behavioral preferences in timing) and which are not (e.g., circadian rhythms, genetic factors). Because of the complexity of these types of studies, the timing of phenotypic assessments and biological sampling (particularly the fasting duration and the time of day) must be carefully considered when drawing conclusions from these investigations.

#### Gut microbiome

The gut is inhabited by trillions of bacteria and other microorganisms which are increasingly recognized as significant contributors to human health. Disturbances to the gut microbiota (i.e., dysbiosis) have been associated with a wide variety of age-related health conditions including bowel, pulmonary, neurologic, skeletal, metabolic, and autoimmune diseases [87–89]. In addition, shifts in the gut microbiota have been linked mechanistically to physiological hallmarks of aging, including chronic inflammation [90], genomic instability [91], mitochondrial dysfunction [92], reduced proteostasis [93], and epigenomic modifications [91]. Thus, the gut microbiome may be an important target for addressing and understanding age-related conditions.

Evidence indicates that advanced age is associated with robust shifts to the microbial composition and function [94]. For example, a recent study indicated that aging individuals who maintain high levels of the taxa *Bacteroides* sp. into later life suffer from increased rates of physical frailty and death [95]. Conversely, the same study reported that individuals undergoing “healthy aging” (determined by physical function measures—e.g., walking speed) possess unique “microbiome signatures” that are linked to health benefits [95]. In fact, this measure of microbial uniqueness was found to be a strong, independent predictor of faster walking speed and lower indices of physical frailty [95], indicating that the microbiome likely plays an intricate role in modifying physical function during aging.

Exercise itself is also a potential modifier to the microbiome during aging. While no studies have thoroughly addressed how exercise modifies the microbiome in an aged population, data indicate that higher physical activity impacts gut microbiome composition and function in adult populations [96–98]. Data have demonstrated that exercise increases the capacity for microbial production of microbial metabolites, including short chain fatty acids (SCFAs) [99, 100], which have anti-inflammatory, satiety, and insulin-sensitizing effects [101–103]. This degree of shift in microbiota-derived metabolites may have a significant impact on metabolic health outcomes, highlighted by recent work indicating that exercise-induced increases in SCFAs have been mechanistically linked to improvements in insulin sensitivity in pre-diabetic subjects [100].

The gut microbiota may also be a tool for understanding fitness and response variability to exercise interventions. For example, studies have shown that the composition and metabolic capacity of the microbiota is a strong predictor of cardiorespiratory fitness (VO_2_max) in adults [98]. In addition, shifts in microbiota-derived metabolites (SCFAs and GABA) were highly predictive of improvements to insulin sensitivity in pre-diabetic adults that underwent a 12-week exercise bout [100]. Meanwhile, other studies have shown that obese individuals exhibit blunted shifts in fecal SCFA compared to their lean counterparts in response to a 6-week aerobic exercise training intervention [96]. Together, these data indicate that the gut microbiota is highly individualized and contributes to response variability to exercise interventions in humans.

#### Knowledge gaps

Can the gut microbiome and its metabolites be used as a predictor of healthy aging? Is an “aged microbiome” less resistant (or more) than a “young” microbiome? Future studies may consider characteristics of the gut microbiota and how it responds to an exercise intervention when trying to understand exercise response variability in an aging population.

### Brain, cognition, and sleep

Aging results in overall declines in many aspects of cognitive function, including episodic memory, executive function, and processing speed [104]. Aging is also linked with an increased risk of various cognitive disorders and neurologic diseases (e.g., Alzheimer’s disease, dementia). Even prior to the onset of clinical disease, the rates of cognitive decline in the USA are substantial, with an estimated two out of three Americans suffering from some level of cognitive impairment at the age of 70 which itself is heterogeneous [105]. Beyond the health and emotional consequences, cognitive decline and neurological disease incur heavy financial burdens that are dealt with by both patients and caregivers. In the USA alone, $305 billion in health care costs were spent on Alzheimer’s disease alone in 2020 [106]. Thus, it is vital for researchers and clinicians to optimize strategies that both prevent and combat age-related cognitive decline in a cost effective and individualized manner.

Exercise and higher physical activity levels are strongly associated with improved cognitive function and reduced risk of age-related cognitive decline [104]. This is evidenced by controlled clinical trials that have shown that aerobic exercise training improves cognitive function in aging populations. For example, a 6-month aerobic exercise intervention in 124 adults between 60 and 75 years of age revealed that exercise selectively improved executive memory function [107]. Exercise-induced improvements in cognition may be related to improved connectivity within the default mode network (DMN) [108], a process that has been evidenced to weaken with age [109]. However, research also indicates cognitive benefits with exercise training are highly variable within an aging population. While it is clear that exercise has widespread effects on brain and cognition, we have a poor understanding of the heterogeneity found in both the magnitude and timing of response to exercise. Numerous factors have been shown to relate to cognitive performance, including age, sex distribution, genetic polymorphisms, dietary and sleep patterns, intellectual engagement, socioeconomic conditions, and early life adversity [110–116]. Thus, prospective appropriately designed clinical trials are needed to understand the underlying factors contributing to these variable improvements in cognition in response to exercise.

Sleep duration and quality may be one of the best predictors of cognitive function during aging. A recent meta-analysis revealed that heterogeneity in cognitive decline is best explained by variability in sleep duration and quality [117]. Interrupted neural pathways and morphological changes to brain structure as a result of sleep deprivation leads to learning and memory deficits in aging individuals. Similarly, older adults with dementia have more sleep disturbances, including shorter sleep duration and fragmented sleep [118].

Recently, lower indices of sleep duration and quality were strongly implicated in increased risk of developing Alzheimer’s disease [119]. Sleep duration and quality may also impact the efficacy of an exercise intervention in improving cognition of older adults. Preclinical studies have demonstrated that sleep restriction countered the positive effects of exercise on memory [120]. Sleep restriction has also been shown to limit the beneficial effects of exercise on fatigue in cancer patients. In animal models, the beneficial effects of exercise in reducing polyp formation in colorectal cancer model was reversed by sleep deprivation [121]. Conversely, other studies show that napping after exercise may synergize with sleep to promote memory formation [122]. In summary, sleep is intricately tied to cognitive function, exercise adaptations, and aging biology. Thus, researchers should attempt to carefully control for sleep duration and quality when investigating exercise response heterogeneity in an aging population.

Exercise has widespread benefits on brain and cognition, but response variation is high in aged individuals. Thus, controlling for the extrinsic, environmental factors that are known to regulate cognition and brain health are needed to understand the true mechanisms underlying the beneficial effects of exercise. To accomplish this, researchers should consider the following when designing studies with cognitive function as an outcome: (1) aim for larger sample sizes that include diverse populations (e.g., race, sex, ethnicity); (2) harmonize and carefully report details of exercise protocols (e.g., mode, intensity); (3) collect multiple samples longitudinally within subjects; and (4) develop systematic reporting methods for factors (e.g., sleep, nutrition) that are known to affect cognitive outcomes. In turn, researchers will be able to more effectively and systematically focus on the mechanisms by which an aging brain responds to exercise.

#### Knowledge gaps

More work is needed on the experimental interaction between exercise and sleep in influencing health and several questions remain. For example, to what extent is one better off sleeping or restricting sleep in order to exercise and what factors mediate heterogeneity in the effects of exercise on sleep?

### Alternative study designs

Knowledge gaps regarding exercise response variation in clinical endpoints are likely due, at least in part, to study design challenges. As discussed above, technical and measurement error inherent to experimentation complicate our ability to realistically quantify real and meaningful biological variation. Additionally, studies that can effectively detect response variation with adequate power often require prohibitively large sample sizes, and often times the rate of the response is completely ignored. Nonetheless, there are approaches that might partially address these issues and thus warrant consideration. For example, phenotyping participants at baseline to delineate subgroups would enable a priori hypothesis testing related to baseline characteristics of those with the most potential to benefit from an exercise intervention. Study designs that progress beyond the randomized controlled trials are currently being implemented, and they may have a place in the study exercise response heterogeneity. Below we discuss four study design methods that have gained traction in the personalized medicine field and thus may be adapted in studies assessing exercise response variation in an aging population.

#### RCT pooling

Treatment response heterogeneity can be defined with four primary components: (1) variation between treatments averaged over all patients, (2) variation between patients given the same treatment, (3) the extent to which the effects of treatments vary from patient to patient, or (4) variation over time when the same patient is given the same treatment. Addressing all the possible sources of heterogeneity is challenging, and the common approach—subgroup analysis—is usually underpowered and can easily lead to misinterpretation of findings. One of the primary benefits of RCT pooling is that researchers can examine a broader representation of populations (ethnicity, sex, underlying health status) than any one trial might offer. RCT pooling also provides more power to investigate interaction terms and subgroup variances that would be challenging to estimate in a single trial.

Pooling RCTs has major challenges as well. First, researchers need access to individual participant data from all relevant clinical trials, which is often prohibitively burdensome. Also, researchers need access to detailed knowledge of each intervention’s study design to account for differences across trials. This includes knowledge of treatment modalities, dose, setting, duration, level of compliance, intervention targets, and the types and characteristics of the participants (e.g., appropriate balance of racial or ethnic groups and gender) that could be relevant to treatment effects. Downstream, “data harmonization” represents another challenge. The process of unifying disparate data fields, formats, dimensions, and columns into an aligned data set can pose serious challenges. Variance differences as well as differences in outcomes can add to the complexity of harmonizing data across RCTs. Assumptions and compromises are an unavoidable part of the data harmonization process and should be implemented carefully with pooled RCTs.

#### Multiphase optimization strategy (MOST)

MOST is an alternative to the classical approach to intervention development and evaluation. The classical approach emphasizes evaluation of the intervention as a treatment package. By contrast, MOST emphasizes building an empirically optimized intervention and only then evaluating it as a package. By optimizing an intervention, we mean achieving a strategic balance of effectiveness, affordability, scalability, and efficiency (*EASE*). MOST relies heavily on gathering information about the individual and combined performance of intervention components by means of highly efficient optimization trials. Possible optimization trial designs include the factorial, fractional factorial, sequential multiple assignment randomized trial (SMART), and micro-randomized trial (MRT). The last two are described below in detail. One objective of optimization may be to reduce variability in response to achieve a good outcome for all intervention participants. MOST may be used to optimize interventions for different settings.

#### Micro-randomization trials (MRTs)

As we move towards precision exercise for an aging population, researchers may also consider implementing a micro-randomized trial (MRT). In an MRT, interventions are randomly assigned many times—often several times per day—over the course of the study period. The timing, randomization probabilities, and the set of allowable interventions in an MRT are allowed to depend on a subject’s evolving health status and situation. Their health status may be comprised of actively collected data such as a brief health inventory, passively collected data such as location, heart rate variability, or blood glucose. The fine granularity of randomization in an MRT allows researchers to study a rich set of scientific queries such as (1) if, when, and for whom an intervention is likely to be effective in the short term; (2) what intervention strategy is optimal for long-term benefit; (3) if and how patients become habituated to treatment; (4) can short-term surrogates predict long-term outcomes; and (5) what factors drive a patient to disengage from treatment. While the set of questions which might be answered through an MRT is vast, each presents its own unique methodological, engineering, and scientific challenges. MRTs typically focus on delivering interventions through an app running on a mobile device. Thus, researchers must invest time and resources into an app which is intuitive and pleasing for the user, does not impose excessive computational or memory demands on a user’s device, and provides adequate protections for a user’s data. In addition, analyzing data from an MRT may require sophisticated statistical methodologies which are only now under development.

#### The sequential multiple assignment randomized trial (SMART) trial design

SMARTs are the gold-standard for data collection when the goal is evaluation of intervention sequences or the development (estimation) of personalized intervention strategies [123–126]. In a SMART, interventions are randomized at key decision points in a patient’s disease progression. SMARTs have been applied to estimate personalized intervention strategies for a wide range of application areas including cancer [127, 128], mental health [129–131], HIV/STI prevention [132], diabetes [133], education [134], and surgery [135]. The set of possible SMART designs are as varied as the diseases and disorders they are used to study; however, many designs are variations of one of the following themes: (R) responder-designs in which patients are randomly assigned treatments in sequence until a satisfactory response is observed; (SU) step-up designs in which low-cost treatments are randomly assigned and then escalated to progressively more costly treatments until a satisfactory response is attained; (SD) step-down designs in which a high-cost treatments are randomly assigned and then responders are randomly de-escalated to less costly treatments. The (R) design reflects the trial-and-error which is common in clinical practice in which a patient’s treatment is adaptively adjusted. The (SU) design is often used in public health interventions when the goal is to identify if and when a patient needs a more expensive treatment. Similarly, the (SD) design is used to identify if and when a high-cost treatment can be discontinued without negatively affecting a patient’s outcome.

#### Personalized (N-of-1) trials

Personalized medicine has as a goal to identify and provide treatments or interventions that best benefit each patient. Randomized controlled trials (RCTs) can provide evidence on how groups of patients respond to a treatment versus how another group of patients respond to a control or to an alternative treatment. However, RCTs do not identify optimal treatments for each individual participant. Moreover, many individuals are excluded from RCTs simply because they do not meet the RCT inclusion/exclusion criteria. RCTs thus can leave major gaps in understanding why individuals respond differently to an intervention. Another approach is personalized (N-of-1) trials, which are defined as “single-participant, multiple-time-period, active-comparator crossover trials that are frequently randomized and can be masked.” This type of randomized controlled trial has as a primary outcome the identification of the statistically superior treatment for one patient, rather than a group of patients, in cases where the optimal treatment is unknown or unclear. Thus, personalized (N-of-1) trials involve switching treatments over time in a defined sequence allowing individual participants to identify how well a given treatment works for them. The use of personalized (N-of-1) trials does require informed selection of treatments, careful implementation, rigorous measurement of outcomes, and proper use of statistical methods. Well-designed personalized (N-of-1) trials assess multiple health outcomes of interest—including exercise level—through a variety of formats, including participant self-report, observed protocol adherence, and remote participant monitoring (RPM) devices. Individual results must be purposefully summarized to convey statistically significant benefits and harms found for each cross-over treatment period. Though important and informative, personalized N-of-1 trials are not without flaws. A major potential criticism for N-of-1 designs in that they frequently rely on participant adherence to data collection and require low levels of missing data. Another challenge is the restriction on the types of conditions which may be evaluated and what types of interventions are used to treat them. Conditions that cannot be reliably measured should be avoided. The study design must also account for any potential treatment carryover effect using planned washout periods. So, some types of exercise types are ideal to study in a personalized trial (e.g., weight training, or HIIT or minimal dose exercise) but other exercise types such as completing a marathon are not ideal. However, when the course of an exercise regimen for an individual participant is uncertain, personalized (N-of-1) trials represent an important tool for clinicians, researchers, and participants to evaluate and identify the most effective exercise intervention for an individual.

## Summary/Conclusion

Exercise is one of the most promising strategies for intervening on the biology of aging. To advance the exercise and aging field, we need to understand how exercise intervenes on the pillars of aging and improves how older adults feel and function. In this regard, a mix of preclinical and translational research models may be appropriate. At present, a central issue complicating our ability to predict the health benefits one might reap from an exercise program is individual response variation in several outcomes.

The NIH NIA Workshop identified the following key knowledge gaps that limit our understanding of heterogeneity of responses to, and optimizing clinical efficacy of, exercise training in older adults: (1) the extent to which exercise training adaptations are impacted by age-related damage is unknown, (2) aging itself is heterogenous contributing to a complex backdrop in which exercise response variation must be interpreted, (3) there is evidence for several intrinsic and extrinsic factors that contribute to exercise response variation in outcomes that are clinically significant for older adults, (4) alternative study designs from the field of personalized medicine should be considered when designing future studies on individual exercise responses, and (5) currently, we have more questions than answers regarding factors that contribute to exercise response variation in older adults. In sum, the optimization of exercise prescription for older adults is ripe for future research. Future innovations may prescreen participants for particular phenotypic or molecular criteria, and then design exercise prescriptions (intensity, duration, frequency, and type) to directly target these age-related deficiencies.
